# From Common Symptom to Critical Diagnosis: Vertigo as a Clue to Thrombotic Thrombocytopenic Purpura

**DOI:** 10.7759/cureus.78305

**Published:** 2025-01-31

**Authors:** Aysche J Stern, Nicholas R Munoz, Reda Khan, Chibuike C Agwuegbo, Salma Yasin, Marie Pearson, Fatima Gauhar

**Affiliations:** 1 Internal Medicine, Temecula Valley Hospital, Temecula, USA; 2 Internal Medicine, Southwest Healthcare, Temecula, USA; 3 College of Medicine, William Jewell College, Liberty, USA; 4 Internal Medicine, Riverside Medical Clinic, Temescal Valley, USA

**Keywords:** acute kidney injury, adamts-13 deficiency, benign paroxysmal positional vertigo, hemolytic anemia, thrombotic thrombocytopenic purpura

## Abstract

Thrombotic thrombocytopenic purpura is a hematologic disease with a high mortality rate that affects multiple organ systems. It is caused by a deficiency of a metalloprotease known as the ADAMTS-13 enzyme. Patients can present with fever, hemolytic anemia, thrombocytopenia, kidney injury, and neurological symptoms. Here we present a case of a female with TTP who was initially diagnosed with benign paroxysmal positional vertigo (BPPV). Her initial symptoms were vertigo, blurry vision, and gait imbalance, which were accompanied by elevated total bilirubin, acute kidney injury, thrombocytopenia, and normal hemoglobin. When the patient followed up in her primary care clinic, she was found to have worsening renal function, hemolytic anemia, and severe thrombocytopenia. She was subsequently diagnosed with TTP and referred to the emergency department (ED) where she received emergent treatment with therapeutic plasma exchange (TPE) and later rituximab. This case stresses the importance of considering TTP in patients presenting with symptoms and laboratory values suggestive of the disease, even when anemia is not present.

## Introduction

Thrombotic thrombocytopenic purpura (TTP) is a rare and life-threatening blood disease caused by severe deficiency of a disintegrin and metalloprotease with thrombospondin type I repeats-13 (ADAMTS-13) enzyme, a von Willebrand factor (VWF)-cleaving protease [1]. This disease is characterized by microangiopathic hemolytic anemia (MAHA), thrombocytopenia, and the formation of platelet-rich thrombi that ultimately result in end-organ ischemia primarily affecting the renal, neurological, and cardiac systems. Historically, a pentad of MAHA, thrombocytopenia, fever, and neurological and renal dysfunction was required for diagnosis of TTP. However, current guidelines consider unexplained MAHA and thrombocytopenia to be sufficient criteria for initiating treatment [2]. The case of a patient who was initially misdiagnosed with benign paroxysmal positional vertigo (BPPV) and later found to have TTP is presented.

## Case presentation

Our patient is a female in her 40s with a history of hypertension, hypothyroidism, hyperlipidemia, and diabetes who initially presented to the emergency department (ED) with vertigo, nausea, and subjective gait imbalance. Initial labs showed a hemoglobin of 14.1 mg/dL, 62,000 platelets/uL, serum creatinine of 1.66 mg/dL, and total bilirubin of 2.6 mg/dL. Despite a normal physical exam, she was diagnosed with BPPV and discharged home. As her symptoms failed to improve, she presented to her primary care physician (PCP) three days later, endorsing vertigo, blurry vision, gait imbalance, nausea, tinnitus, and bilateral leg weakness. She further reported non-bloody diarrhea and vomiting for three days. The family history was negative for cancer, and she denied any smoking and illicit drug or alcohol use. The physical exam was normal other than a slow gait, without objective gait imbalance. A computerized tomography (CT) scan of the head showed no acute abnormalities but repeat laboratory work was significant for hemoglobin of 8.8 mg/dL, 15,000 platelets/uL, serum creatinine of 4.87 mg/dL, total bilirubin of 2.3 mg/dL, direct bilirubin of 0.6 mg/dL, and AST of 45 IU/L, as well as schistocytes on blood smear. Her PLASMIC score was calculated as six indicating a 72% risk for ADAMTS-13 deficiency. Together, these signs of worsening kidney injury with hemolytic anemia, thrombocytopenia, and neurological symptoms strongly suggested the diagnosis of TTP, and the patient was advised to immediately present to the ED. 

After a presentation to the ED, she was admitted to the ICU, where follow-up laboratory analysis showed creatinine of 4.4 mg/dL, platelets of 6,000 platelets/uL, hemoglobin of 7.4 g/dL, negative indirect and direct antiglobulin test, total bilirubin of 1.9 mg/dL, direct bilirubin of 0.6 mg/dL, D-dimer of 20.8 ug/mL, elevated reticulocyte count of 3.6%, normal fibrinogen at 347 mg/dL, haptoglobin of <10 mg/dL, a normal troponin of 65 ng/L, homocysteine of 13.4 umol/L, methylmalonic acid of 0.22 mmol/mL, and lactate dehydrogenase of 1,754 U/L (Table 1). Her laboratory values indicated a past Epstein-Barr and cytomegalovirus infection. She tested positive for antinuclear antibody and Sjögren’s-syndrome-related antigen A autoantibodies. A hepatitis panel was negative. Stool studies were not completed as she did not have a bowel movement during hospitalization. 

**Table 1:** The patient’s lab values on admission to the intensive care unit.

Test	Result	Normal Value
White Blood Cell Count	7,600 cells/uL	4,500-11,000 cells/uL
Hemoglobin	7.4 g/dL	12.0-16.0 g/dL
Hematocrit	21.3%	36-46%
Platelets	6,000 platelets/uL	150,000-400,000 platelets/uL
Blood Urea Nitrogen	63 mg/dL	8-20 mg/dL
Creatinine	4.4 mg/dL	0.6-1.2 mg/dL
Alkaline Phosphatase	48 IU/L	45-115 IU/L
Aspartate Aminotransferase	36 IU/L	8-40 IU/L
Alanine Transaminase	21 IU/L	8-40 IU/L
Reticulocyte Percentage	4.10%	0.5-1.5 %
Fibrinogen	347 mg/dL	150-350 mg/dL
Haptoglobin	<10 mg/dL	50-150 mg/dL
Lactate Dehydrogenase	1754 U/L	40-90 U/L
Direct Bilirubin	0.6 mg/dL	0.0-0.3 mg/dL
Total Bilirubin	1.9 mg/dL	0.1-1.0 mg/dL
D-Dimer	20.8 mg/L	<0.5 mg/L
Troponin	65 ng/L	<14 ng/L
Homocysteine	13.4 mmol/mL	0.41-1.89 mmol/mL
Methylmalonic Acid	0.22 mmol/mL	150-370 mmol/mL
ADAMTS-13	<5%	40-140%

Due to high suspicion for TTP, treatment with therapeutic plasma exchange (TPE) with fresh frozen plasma transfusion in addition to prednisone 1 mg/kg daily was initiated despite pending results of the ADAMTS-13 activity testing. After five days of TPE, the patient's hemoglobin and platelet levels stabilized, and she was discharged with instructions to continue twice weekly complete blood counts until she could establish care with a hematologist. Her ADAMTS-13 activity assay resulted as <5%, with a normal ADAMTS-13 genotype confirming the diagnosis of acquired TTP.

At her outpatient follow-up after initial hospitalization, the patient reported normal bowel movements but continued to endorse balance issues and blurry vision. Laboratory values at this time resulted in a hemoglobin of 6.5 mg/dL, a platelet count of 11,000 platelets/uL, and a serum creatinine of 2.06 mg/dL, suggesting a relapse of TTP. She was hospitalized for eight days when she went into second remission. During this hospitalization, she received additional TPE and was started on prednisone 80 mg daily. After her platelets increased to greater than 11,000 platelets/uL, she was started on rituximab infusions and discharged.

However, after discharge, the patient was unable to receive rituximab on an outpatient basis and was ultimately hospitalized a third time for rituximab infusions. This hospitalization was complicated by liver injury with AST and ALT peaking at 927 U/L and 837 U/L respectively, and rituximab was subsequently discontinued. She underwent a liver biopsy showing sinusoidal dilation and edema with sparse portal inflammation without fibrosis or cirrhosis. Given those nonspecific findings, rituximab was assumed as cause of this iatrogenic liver injury. 

**Figure 1:**
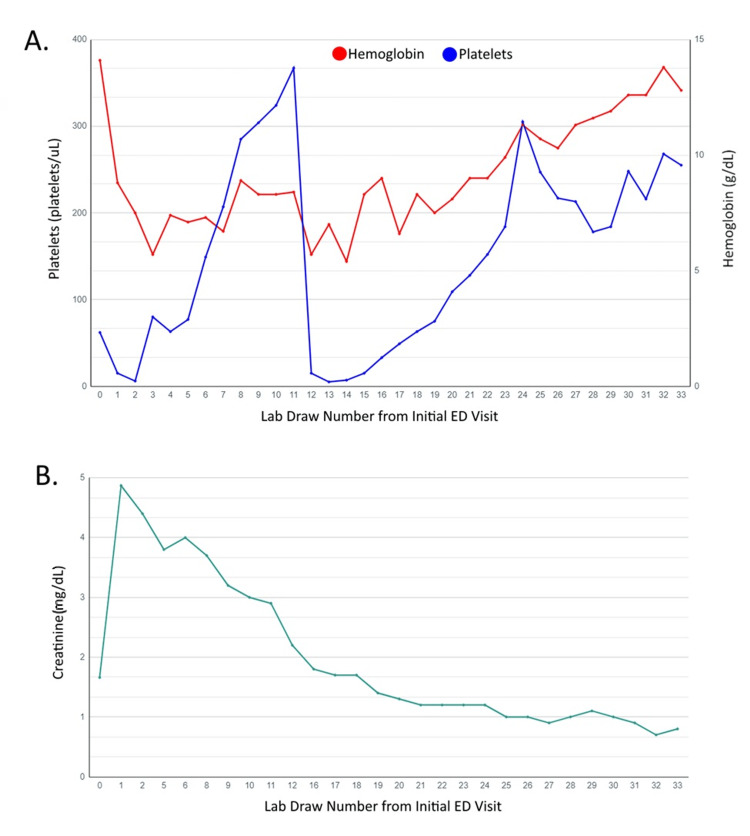
Graph of the patient's platelet count (A), hemoglobin level (A), and creatinine level (B) starting from the initial visit to the emergency department. Blood draw 0: initial visit; blood draw 1: during follow-up with primary care; blood draw 2-11: intensive care unit admission; blood draw 12: post-hospital follow-up with primary care; blood draw 13-23: third hospitalization; blood draw 24-33: follow-up labs after the patient’s third hospitalization

**Figure 2:**
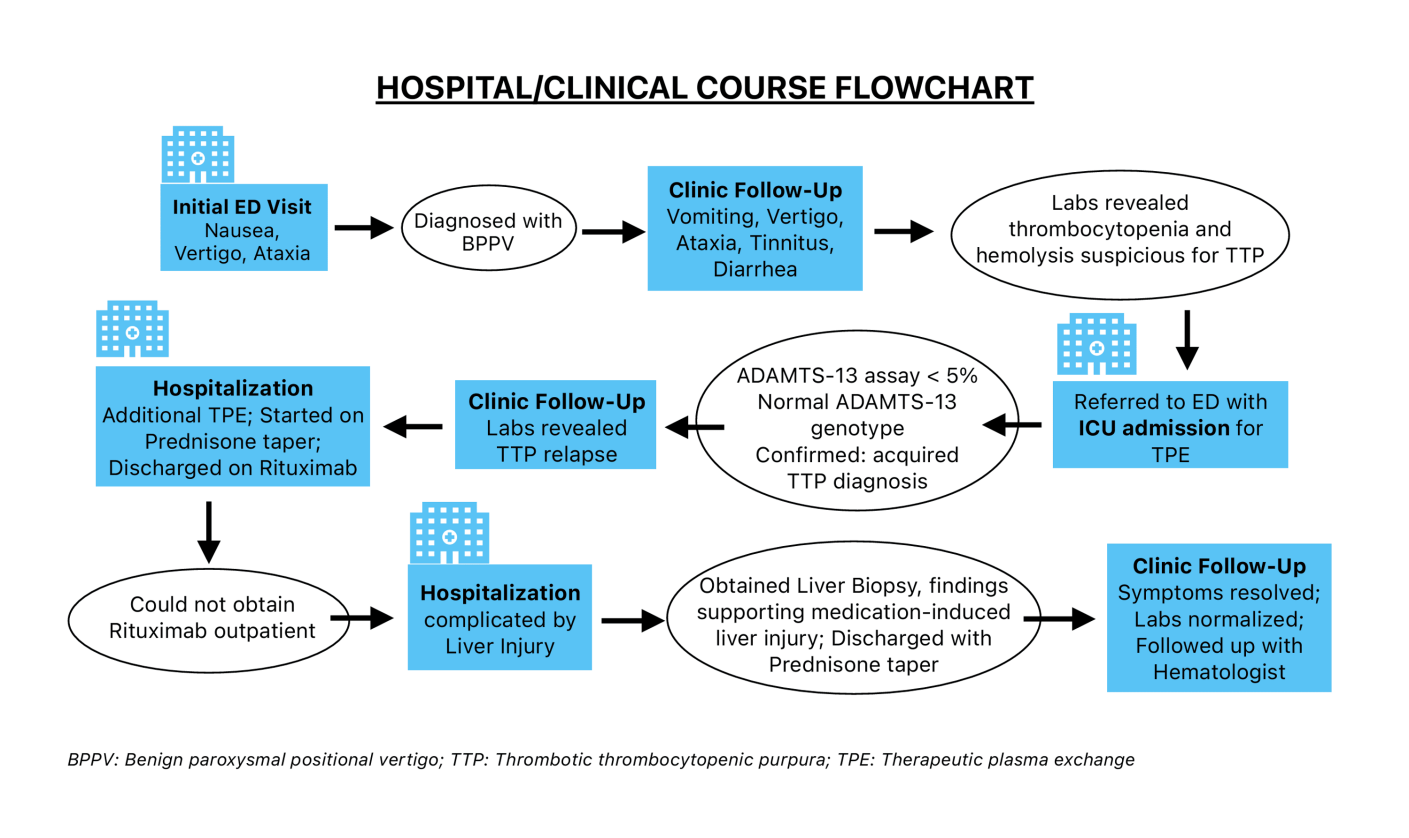
Flowchart of the patient's clinical course. Figure courtesy of Salma Yasin.

Outpatient follow-up showed stable laboratory values including a normal hemoglobin level, platelet count, and creatinine, with no further relapse thereafter (Figure 1). The patient reported that her balance had improved; however, she continued to complain of dizziness and blurry vision. She denied symptoms suggestive of autoimmune disease such as dry eyes, dry mouth, rashes, joint pain, and muscle pain. Repeat ADAMTS-13 levels were normal two months after her initial ICU stay. Her liver function tests normalized, and she restarted her home medications metformin and rosuvastatin. Her symptoms resolved, allowing her to start exercising and she returned to work. She continues to follow up with her hematologist and PCP for regular check-ups (Figure 2). 

## Discussion

The classic pentad of symptoms in TTP is only present in 7% of patients [3]. Neurological symptoms are present in 40-80% of patients, 46% of patients have signs and symptoms of bleeding, and 30-40% of patients have gastrointestinal symptoms [3,4]. TTP has a median onset during the fourth decade of life. It is more common in females and African American patients [5].

**Figure 3:**
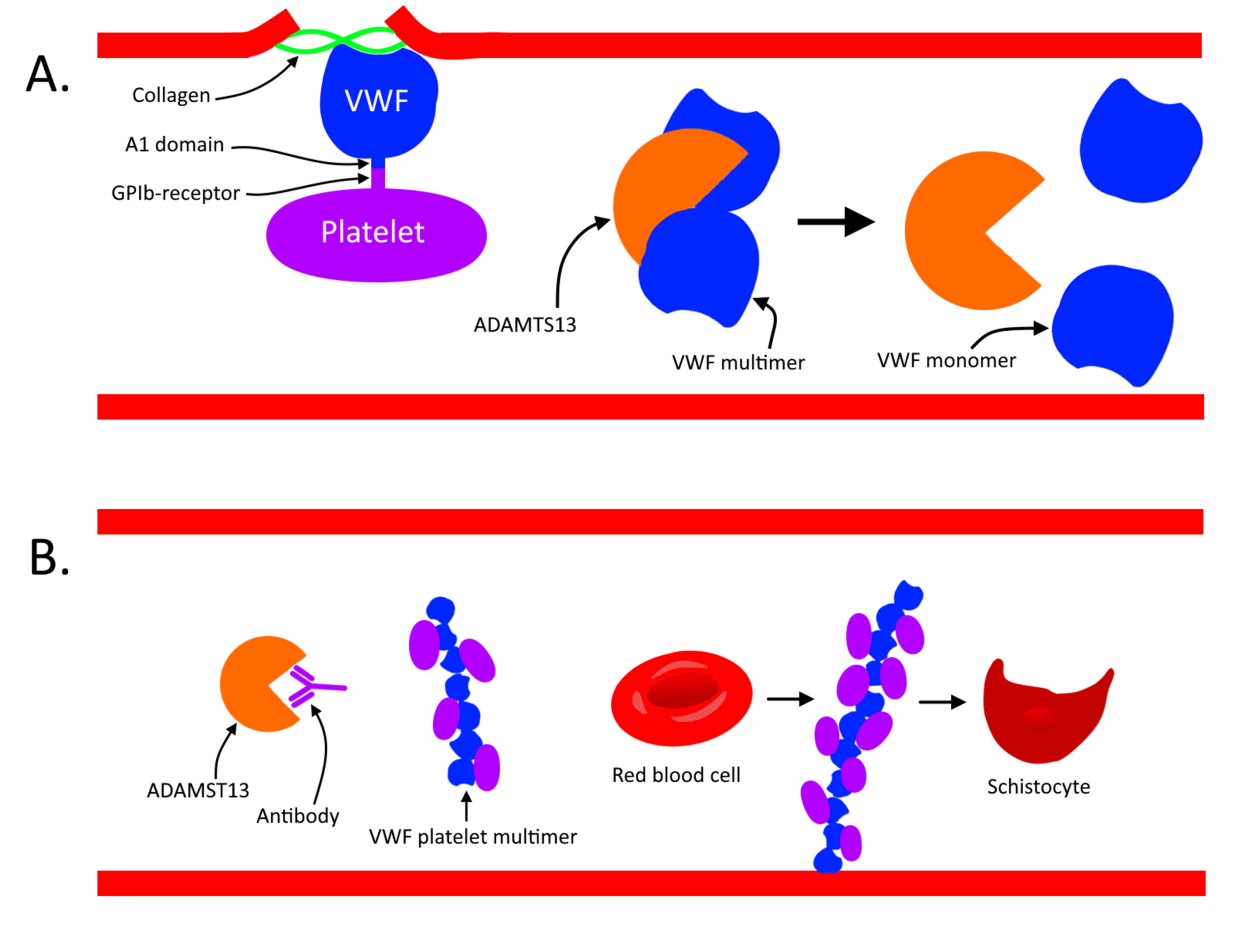
A. Normal physiology of disintegrin and metalloprotease with thrombospondin type 1 repeats-13 (ADAMTS-13), which cleaves von VWF multimers into monomers. During normal hemostasis, the VWF A1 domain binds to the platelet GP1b receptor, facilitating platelet adhesion. B. Pathophysiology of TTP. Antibodies targeting ADAMTS-13 block its enzymatic activity, leading to the accumulation of VWF-platelet multimers. These multimers cause red blood cell lysis and the formation of schistocytes. Additionally, the multimers may embolize to arterioles, causing ischemia in affected organs. TTP, thrombotic thrombocytopenic purpura; VWF, von Willebrand factor; GP1b, glycoprotein 1b Figure courtesy of Nicholas Munoz.

Many of the typical symptoms found in TTP are caused by the embolization of platelet-rich thrombi to arterioles. During normal primary hemostasis, damage to the blood vessel wall causes bleeding and exposure of subendothelial collagen. VWF sequesters platelets to this exposed collagen and other proteins to achieve hemostasis. This interaction is mediated by the VWF A1 domain binding to the platelets GP1b receptor (Figure 3A) [6]. In TTP, deficiency of ADAMTS-13 causes accumulation of ultra-large von Willebrand factor (ULVWF) multimers and platelet aggregation, leading to the formation of thrombi and ultimately MAHA (Figure 3B) [2]. Causes are divided into two groups: acquired (>90% of cases) and congenital. In acquired disease, the development of autoantibodies is most commonly idiopathic (primary) but can also be associated with underlying disease processes (secondary) like bacterial infections and autoimmune diseases [1]. Other risk factors include pregnancy, human immunodeficiency virus infection, acute illnesses, or drugs [1]. In 10% of cases, autoimmune diseases later develop after a previous episode of TTP [1]. 

The appropriate workup for TTP includes a complete blood count with a smear, a complete metabolic panel, and an ADAMTS-13 level [3]. The PLASMIC score is a seven-component score used to risk stratify patients for the disease. An ADAMTS-13 level of <10% supports the diagnosis [3]. MAHA is diagnosed by schistocytes on blood smears and laboratory findings such as elevated lactate dehydrogenase, decreased haptoglobin, and elevated bilirubin [7]. 

Without treatment, TTP has a survival rate of about 10%. TPE increases the survival rate by 78% [8]. Treatment consists of TPE, rituximab, corticosteroids, and caplacizumab as this combination has been shown to reduce mortality [9]. TPE removes anti-ADAMTS-13 antibodies and replaces ADAMTS-13. Corticosteroids and rituximab suppress the immune response against ADAMTS-13, helping to maintain remission and reduce relapses [10]. The early use of rituximab with TPE has been shown to prolong the duration of remission. Caplacizumab is an anti-VWF antibody that prevents the interaction of VWF with the platelet glycoprotein Ib-IX-V receptor, decreasing microvascular thrombosis. Trials have shown that, compared to patients in the placebo group, patients who received caplacizumab had a shorter time to normalization of platelets, lower mortality and recurrence rates, and fewer major thromboembolic events [11]. For patients in remission with evidence of low plasma ADAMTS-13 activity, the use of rituximab has been shown to decrease relapses [12]. 

ADAMTS-13 activity <10% is highly predictive of relapse, maintaining activity above that level can prevent relapses [13]. If a patient's ADAMTS-13 activity is persistently <10%, rituximab therapy can prevent a relapse [14]. During remission, serial ADAMTS-13 levels should be monitored in addition to chemistry panels, complete blood counts, and LDH. After an acute episode, ADAMTS-13 levels can be measured monthly for three months, every three months for one year, then once or twice a year if the patient is stable [15].

At the time of her initial evaluation in the ED, our patient demonstrated some signs of TTP including vertigo, acute kidney injury, moderate thrombocytopenia, and elevated bilirubin levels. The diagnosis was likely missed because of normal hemoglobin levels. The subsequent laboratory values in the outpatient setting were more significant and, mainly due to obvious signs of hemolytic anemia, indicated TTP, which required immediate hospitalization. Further investigations revealed a positive antinuclear antibody and Sjögren’s-syndrome-related antigen A; however, she did not meet diagnostic criteria for either Sjögrens syndrome or lupus. While the cause of her TTP remains unclear, an underlying autoimmune disorder may have played a role. 

## Conclusions

TTP is a rare but life-threatening disease, and both prompt recognition and treatment are important to reduce the associated high mortality rate. This case is unique due to the absence of anemia despite other typical signs and symptoms like vertigo, acute kidney injury, thrombocytopenia, and elevated bilirubin levels at the initial presentation. TTP should be considered in patients with a variety of presentations and laboratory values suggestive of the disease, even in the presence of normal hemoglobin levels. TTP treatment relies on TPE, rituximab, caplacizumab, and corticosteroids. Relapse is common, and close outpatient monitoring is recommended.
